# Characterisation of microvessel blood velocity and segment length in the brain using multi-diffusion-time diffusion-weighted MRI

**DOI:** 10.1177/0271678X20978523

**Published:** 2020-12-16

**Authors:** Lauren A Scott, Ben R Dickie, Shelley D Rawson, Graham Coutts, Timothy L Burnett, Stuart M Allan, Geoff JM Parker, Laura M Parkes

**Affiliations:** 1 Division of Neuroscience and Experimental Psychology, School of Biological Sciences, Faculty of Biology, Medicine and Health, University of Manchester, Manchester, UK; 2 Geoffrey Jefferson Brain Research Centre, Manchester Academic Health Science Centre, Northern Care Alliance & University of Manchester, Manchester, UK; 3 The Henry Royce Institute, Department of Materials, The University of Manchester, Manchester, UK; 4 Bioxydyn Limited, Manchester, UK; 5 Centre for Medical Image Computing, Department of Computer Science and Department of Neuroinflammation, University College London, London, UK

**Keywords:** Blood velocity, diffusion time, intravoxel incoherent motion, microvessel structure, velocity autocorrelation

## Abstract

Multi-diffusion-time diffusion-weighted MRI can probe tissue microstructure, but the method has not been widely applied to the microvasculature. At long diffusion-times, blood flow in capillaries is in the diffusive regime, and signal attenuation is dependent on blood velocity ( v ) and capillary segment length ( l ). It is described by the pseudo-diffusion coefficient ( $D^{*}=vl/6$ ) of intravoxel incoherent motion (IVIM). At shorter diffusion-times, blood flow is in the ballistic regime, and signal attenuation depends on v , and not l . In theory, l could be estimated using $D^{*}$ and v . In this study, we compare the accuracy and repeatability of three approaches to estimating v , and therefore l : the IVIM ballistic model, the velocity autocorrelation model, and the ballistic approximation to the velocity autocorrelation model. Twenty-nine rat datasets from two strains were acquired at 7 T, with b -values between 0 and 1000 smm^−2^ and diffusion times between 11.6 and 50 ms. Five rats were scanned twice to assess scan-rescan repeatability. Measurements of l were validated using corrosion casting and micro-CT imaging. The ballistic approximation of the velocity autocorrelation model had lowest bias relative to corrosion cast estimates of l , and had highest repeatability.

## Introduction

Damage to the cerebral microvasculature may contribute to the development and progression of several neurological disorders, including cerebral small vessel disease and Alzheimer’s disease.^1^ There is evidence for thin and fragmented vessels, reductions in capillary density, abnormal branching and increased vessel tortuosity in Alzheimer’s disease and other dementias.^1–6^ Jespersen and Østergaard^7^ suggest such morphological changes may alter capillary transit time heterogeneity which may limit the extraction efficacy of oxygen into tissue, impeding neural function. However, morphological changes may also be compensatory. For example, changes (e.g. angiogenesis) resulting in collateral flow^8^ may tend to restore oxygen levels to normal. The nature and timing of these microvessel changes is unclear – questions remain as to whether changes precede or develop alongside neurodegeneration and whether they are detrimental or compensatory. Novel methods for non-invasively measuring microvessel structure *in-vivo* will help address these questions.

**Figure 1.**
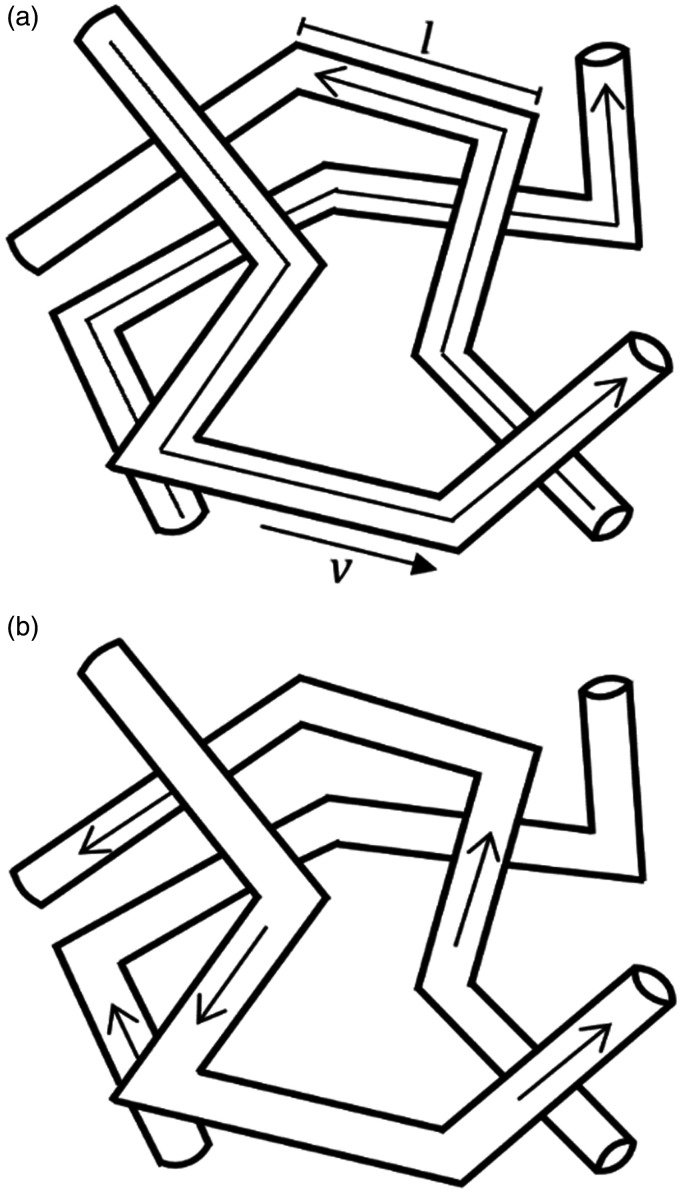
Blood flow in a capillary network with capillary segment length,  l , and average blood velocity, v : isotropic incoherent motion in the diffusive regime with long diffusion-times in (a) and isotropic coherent motion in the ballistic regime with short diffusion-times (b). This image was adapted from Le Bihan et al. (10).

Varying the magnitude and timing of diffusion gradients in diffusion-weighted (DW) magnetic resonance imaging (MRI) allows access to, and isolation of, the extravascular and intravascular components of the DW-MRI signal. Most attention to date has focussed on modelling the extravascular component of DW-MRI signals with the aim of estimating cell size, density, and membrane permeability.^9^ The theory of intravoxel incoherent motion (IVIM)^10–13^ first modelled the capillary contribution to the DW signal in two flow regimes (Figure 1). In the diffusive regime (Figure 1(a)), blood flow is assumed to change direction numerous times during the diffusion-time. The signal attenuating effects of this motion are characterised by a pseudo-diffusion coefficient ( $D^{*}$ ) where $D^{*}=vl/6$ ( v is the average blood velocity and l is the average microvessel segment length). The ballistic regime (Figure 1(b)) describes the other limiting case, in which blood flow does not change direction during the diffusion-time. The signal attenuating effects are described by a sinc function which is dependent on v , but independent of l. ^10^

In theory, microvessel structure could be determined using the two IVIM flow regimes. Fitting the ballistic regime model to data acquired at low diffusion-times (estimating v ), and the diffusive regime model to data acquired at longer diffusion-times (estimating $D^{*}$ ) allows l to be derived.^10^ However, Le Bihan states that the sinc behaviour has never been reported experimentally^12^ and some research suggests a higher order IVIM model may be favourable,^14–18^ particularly at low diffusion-times.^19^ Kennan et al.^20^ introduced an alternative model of signal attenuation in microvessels based on velocity-autocorrelation (VA) theory. The VA model is diffusion-time dependent, so is valid between the two IVIM regimes, and reduces to the IVIM model in the diffusive regime. However, the model describes signal attenuation in the ballistic regime as a mono-exponential decay, rather than a sinc function.

Approaches to model the intravascular component are growing and recent efforts have focussed on using flow-compensated gradients^15^,^21–23^ at a single or multiple diffusion-times. Flow-compensated gradients rephase signal loss from ballistic flow (i.e. that from spins undergoing zero direction changes in the diffusion-time), leaving only attenuation due to spins that change direction. Acquiring data at multiple diffusion-times enables estimation v and l. ^23^ However, flow-compensation doubles the minimum diffusion-time, limiting the ability to probe the smallest vessels, and lengthens the minimum echo time. Data acquired using non-flow compensated gradients (i.e. bipolar gradient) are also sensitive to v and  l , and can be readily acquired on most commercial scanners.

In this paper, we show that accurate and precise estimates of v and l can be obtained using bipolar non-flow compensated gradients. The IVIM model is used to predict signal attenuation at long diffusion-times to estimate $D^{*}$ , and three approaches for estimating v , and therefore l , are evaluated (ballistic IVIM model, velocity autocorrelation model (VA), ballistic approximation of the velocity autocorrelation model (VAB)). Accuracy of microvessel segment length estimates are assessed by comparison with micro-CT images of a microvascular corrosion cast. To assess agreement of measurements between two rat strains, expected to have similar regional variation, regional estimates are compared between two rat strains (Fischer 344 and Wistar-Kyoto). Scan-rescan reproducibility was assessed in a subset of F344 rats. We show the VAB model has the lowest bias relative to corrosion cast estimates, and the highest scan-rescan repeatability of the three methods tested.

## Theory

In the presence of diffusion-sensitising gradients, attenuation of the MR signal can be described by a two-compartment model, describing the intra and extra-vascular contributions:

(1) $$\begin{array}{c} \frac{S\left(b\right)}{S_{0}}=fe^{-bD_{b}}H\left(b\right)+\;\left(1-f\right)e^{-bD}\; \end{array}$$

where $b=\gamma^{2}\delta^{2}G^{2}\left(\Delta -\frac{\delta }{3}\right)$ when employing pulsed gradient spin echo (PGSE) sensitisation. γ is the proton gyromagnetic ratio; G , the magnitude of the diffusion gradient pulses; δ , the duration of each diffusion gradient pulse and; Δ , the diffusion-time, the time between the gradient pulses. S(b) is the DW-MR signal as a function of b ; H(b) , the attenuation due to flowing spins as a function of b -value; $S_{0}$ , the signal at b=0 ; D , the extravascular diffusion coefficient and; f , the perfusion fraction.^24^ $D_{b}$ is the apparent diffusion coefficient of water in blood.^12^ The extra vascular contribution is modelled as a monoexponential since the highest b -value used ( b = 1000 smm^−2^) is below the threshold at which one sees biexponential signal attenuation due to fast and slow compartments.^25^

### Intravoxel incoherent motion model

The IVIM model^12^ has solutions for the diffusive and ballistic regime. In both regimes, IVIM assumes the pseudo-random orientation of capillaries. In the diffusive regime, blood in capillaries is assumed to change segment, and so direction, many times during the diffusion-time. Thus blood flow is described as pseudo-diffusion. The time between directional changes of blood flow ( $T_{0}$ , also termed correlation time) is assumed to be much shorter than the diffusion-time ( $T_{0}<\Delta$ ). The intravascular signal is described by the exponent of a fixed parameter, independent of Δ , which is termed the pseudo-diffusion coefficient, $D^{*}$ :

(2) $$\begin{array}{c} H=e^{-bD^{*}}=e^{-b\frac{vl}{6}}\; \end{array}$$

where v is the average blood velocity and l is the average vessel segment length. In theory, l could be estimated using equation (2) and estimates for $D^{*}$ and v . We consider three methods to estimate v , and therefore l :

### Method 1. IVIM (ballistic regime)

In the ballistic regime ( $\Delta <T_{0}$ ), blood is assumed not to change direction during the diffusion-time. The signal attenuation is then only dependent on v , not l , and is modelled by a sinc function:^10^

(3) $$\begin{array}{c} H=\text{s}\text{i}\text{n}\text{c}\left(cv\right)\; \end{array}$$

where $c=\gamma \left(\int_{0}^{TE/2}-Gt\text{d}t+\int_{TE/2}^{TE}Gt\text{d}t\right)$ ^19^ which is equivalent to $c={\Delta \;\left(b/\left(\Delta -\delta /3\right)\right)}^{\frac{1}{2}}$ for the bipolar gradients used in this study.

### Method 2. Velocity Autocorrelation model

At intermediate diffusion-times, where the diffusion-time may be of the order of $T_{0}$ ( $\Delta ∼T_{0})$ , the signal attenuation due to blood flow is diffusion-time dependent and H is given by the velocity autocorrelation model of Kennan et al.:^20^

(4) $$\begin{array}{c} H=\exp\left(-b\frac{\Omega v^{2}T_{0}}{3\delta^{2}\left(\Delta -\frac{\delta }{3}\right)}\right)\; \end{array}$$

where:

(5) $$\begin{array}{c} \Omega =\delta^{2}\left(\Delta -\frac{\delta }{3}\right)-2T_{0}^{2}{\delta -T}_{0}^{3}q \end{array}$$

(6) $$\begin{array}{c} \begin{array}{l} q=2\text{e}\text{x}\text{p}\left(-\frac{\Delta }{T_{0}}\right)+2\text{e}\text{x}\text{p}\left(-\frac{\delta }{T_{0}}\right)-\text{e}\text{x}\text{p}\left(-\frac{\Delta +\delta }{T_{0}}\right)\\ \;-\text{e}\text{x}\text{p}\left(-\frac{\Delta -\delta }{T_{0}}\right)-2 \end{array} \end{array}$$

In this model, two parameters must be estimated ( v and $T_{0}$ ), in contrast to the ballistic models where only v is estimated.

In the limit of the diffusive regime $\;(T_{0}<\Delta$ ), H reduces to the IVIM model of pseudo-diffusion and is equal to equation (2).

### Method 3. Velocity autocorrelation model (ballistic regime)

In the limit of the ballistic regime $\;(T_{0}>\Delta$ ), the velocity autocorrelation model remains exponential with  H :

(7) $$\begin{array}{c} H=\exp\left(-b\frac{v^{2}\Delta^{2}}{6(\Delta -\delta /3)}\right) \end{array}$$

Neil and Ackerman showed in 1992^14^ that the intravascular compartment may not be a monoexponential decay. This theory is also discussed by Henkelman et al.^18^ who describe the flaws associated with the common monoexponential IVIM model which assumes flow in capillaries is contributing to signal attenuation only. The authors suggest a model which reflects the hierarchical structure of the vessel network, and thus includes contribution from larger vessels. More recently, a more general approach has been taken to characterise the contribution of larger vessels. Fournet et al.,^19^ Wu et al.^15^ and others^16^,^17^ use a two compartment intravascular model representing ‘fast’ and ‘slow’ perfusing components of the signal rather than specifically describing veins and arteries as in Henkleman’s model.^18^ The slow compartment may correspond to pseudo-diffusive flow and the fast, ballistic flow, as described by Wu et al.^15^ Unfortunately, due to limitations regarding the number of b -values, it was not justified to investigate these models in this work. The model suggested by Henkelman et al.^18^ presents an interesting alternative method of modelling the signal attenuation. However, since it does not allow for estimation of v , and thus l , which is the main aim of this work, it is not used here. Here, we introduce sensitivity to v through manipulation of the diffusion-time at fixed b -values.

## Materials and methods

Multi-diffusion-time DW-MRI data is acquired in two strains of rat in order to estimate $D^{*}$ , v , and l using equations (2) to (7). To determine the optimal method for estimating v , and therefore l , the accuracy of estimates from each method are evaluated against measurements obtained from x-ray CT images of a vascular corrosion cast. The scan-rescan repeatability of estimates from the two most accurate methods are compared, in addition to a regional comparison of parameter estimates between the two rat strains.

### MRI acquisitions

**Figure 2.**
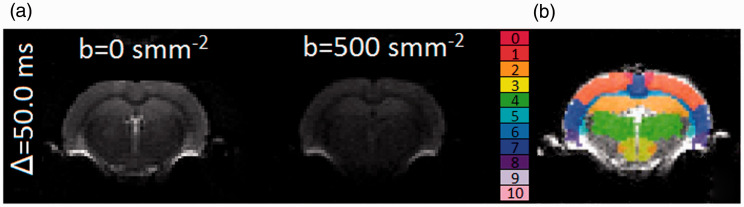
Axial mid-brain slice DW-MR image across two b-values and one diffusion-time (a), region-of-interest (ROI) map, from the Schwarz et. al^27^ rat atlas, overlaid on an axial mid-brain slice DW-MR image (b). Region 0: motor cortex; 1: parietal cortex; 2: hippocampus; 3: hypothalamus; 4: thalamus; 5: striatum; 6: corpus callosum; 7: temporal cortex; 8: entorhinal cortex; 9: cingulate cortex; 10: frontal cortex.

MRI scanning was performed on a Bruker Avance III console (Bruker BioSpec, Karlsruhe, Germany) interfaced with an Agilant 7 T 16 cm bore magnet. A Bruker transmit only resonator (T11070V3) was used for transmission and a Bruker rat brain surface coil (T11205V3) was used for reception. To demonstrate robustness of measurements across different strains, two rat strains were scanned: Fischer-344 (F344) (n = 19, 8 male, 11 female) and Wistar-Kyoto (WKY) (n = 10, all male) at 13 months. Five F344 rats were re-scanned within two weeks to investigate scan-rescan repeatability of measures. The animals were anaesthetised with 2% isoflurane and 100% O_2_ for the duration of the scans. High resolution *T_2_*-weighted RARE images were acquired for regional segmentation purposes (see later). The following parameters were used: TR = 3188 ms, TE = 11 ms, voxel size 0.11 × 0.1 × 1 mm^3^, RARE factor 8, NSA = 2. DW-MR images (Figure 2(a)) were obtained for each rat along three orthogonal diffusion-encoding directions. The b -value was varied through eight values: 0, 10, 20, 50, 100, 200, 500 and 1000 s mm^−2^. For each b -value, the diffusion-time ( Δ ) was varied through four values: 11.6, 20, 40, 50 ms. The lowest diffusion-time chosen was the lowest possible given the scanner maximum gradient strength (*G*_max_ = 375 mTm^−1^) and slew rate (3375 Tm^−1^s^−1^). To ensure the b -value remained fixed while changing diffusion-time, the amplitude of the diffusion gradient pulse, G , was altered while fixing the gradient duration,  δ . Other sequence parameters were: TR = 3000 ms; TE = 66.86 ms; voxel size = 0.313 × 0.313 × 1 mm^3^; δ = 5.8 ms. MRI data is available upon reasonable request to the corresponding author.

Animal experiments were reviewed and approved by the Animal and Welfare Ethical Review Board of the University of Manchester and performed under appropriate Home Office authority in line with the Animals (Scientific) Procedures Act 1986. This work is in compliance with ARRIVE guidelines (Animal Research: Reporting in Vivo Experiments). Breeding, housing, and husbandry details, conforming to the ARRIVE reporting guidelines^26^ can be found in supplementary materials.

### Extraction of regional MRI signals

Segmentation of key brain regions was performed by registering the reference *T*_2_-weighted image from the Schwarz et al.^27^ rat atlas (Figure 2(b)) to the acquired *T*_2_-RARE image using the Insight Toolkit (ITK) within the Advanced Normalisation Tools (ANTs) package (version 2),^28^ and applying the estimated transform parameters to the label image. MRI signals from the following regions were extracted: striatum, temporal cortex, cingulate cortex, entorhinal cortex, frontal cortex, motor cortex, somatosensory cortex, hippocampus, hypothalamus and thalamus. Signal from each of the three orthogonal diffusion directions were averaged (using the mean). The voxel-wise signal to noise ratio (SNR) of normalised DW-MR data was estimated at b = 10 smm^−2^ using equation (A7) of Dietrich et al.^29^ for each region using repeat F344 rat data. The various models were then fit to the average signal from each region, as described in the next section, in order to extract estimates for $D^{*}$ , v and l for each rat. L.S. performed data processing and was blinded to rat strain.

### Model fitting

#### Estimation of D and f

The extravascular component of the DW signal was estimated using high b -value data ( b = 500 smm^−2^ and b = 1000 smm^−2^) for which the contribution of the intravascular component is considered negligible ( ∼ 0.2% if f=0.08 , $D=0.8\times 10^{-4}$ mm^2^ s^−1^ and $D^{*}=0.8\times 10^{-3}$ mm^2^ s^−1^). This ‘two-step’ method^30^ is commonly used across IVIM studies.^12^,^19^,^31^,^32^ The extravascular diffusion coefficient, D , was estimated for each diffusion-time separately using:

(8) $$\begin{array}{c} D=\frac{1}{b_{1}{-b}_{2}}\text{l}\text{n}\left(\frac{S_{2}}{S_{1}}\right) \end{array}$$

The perfusion fraction, f , was then estimated for each diffusion-time using:

(9) $$\begin{array}{c} f=1-\frac{S}{S_{0}}e^{bD} \end{array}$$

#### Estimation of intravascular parameters

The extravascular component is subtracted from the total normalised DW signal and divided by f to isolate the intravascular component:

(10) $$\begin{array}{c} \frac{1}{f}\left(\frac{S}{S_{0}}-\left(1-f\right)e^{-bD}\right)=\;e^{-bD_{b}+d}H\; \end{array}$$

where d is a parameter added to improve fitting. Theoretically d should be equal to zero. However, due to bias from the two-step method and effective b -values, non-zero values of the intercept were observed.

Models of H independent of diffusion-time (equations (2), (3) and (7)) were substituted into equation (10) and data fitted to each diffusion-time separately. The VA model (equation (4)) was fitted across all diffusion-times simultaneously. Data with b < 500 smm^−2^ was used and model fitting used *‘lsqcurvefit’* in Matlab (R2018a) with a trust-region-reflective algorithm. $D_{b}$ was set to equal $1.75\times 10^{-3}$ mm^2^ s^−1^.^19^

At high diffusion-times (50 ms), equation (2) is assumed to be valid, allowing for estimation of $D^{*}$ . Estimates from the IVIM ballistic and VA ballistic models were assumed to be valid at Δ  = 11.6 ms (assuming l∼ 30 µm^33^ and v∼ 2 mms^−1^,^34^ $T_{0}$ would be approximately 15 ms).

All models with exception of equation (2) provide an estimate for v . Estimates of l were generated by combining estimates of v with those of $D^{*}$ estimated using the IVIM diffusive model (equation (2)).

#### Evaluating the validity of model approximations

In theory, l , can be estimated from data sets acquired at only two diffusion-times – the first acquired in the diffusive regime to estimate $D^{*}$ (equation (2)) and the second acquired in the ballistic regime to estimate v (equations (3) or (7)). While this approach is attractive as it promises short acquisition time, this method has not been tested *in-vivo* and it is not clear whether the conditions for diffusive and ballistic flow are met. A more general approach would be to fit the VA model (equation (4)), which could circumvent the need to approximate signals and possibly remove bias. Furthermore, data with intermediate Δ values could be included, potentially improving precision of parameter estimates. In order to determine the limit of validity, values for v and l were estimated for each diffusion-time for the VAB model, these values were then compared with those of the VA model. Since the VAB model is a simplification of the VA model, the two should converge where the VAB model is valid.

### Corrosion casting

**Figure 3.**
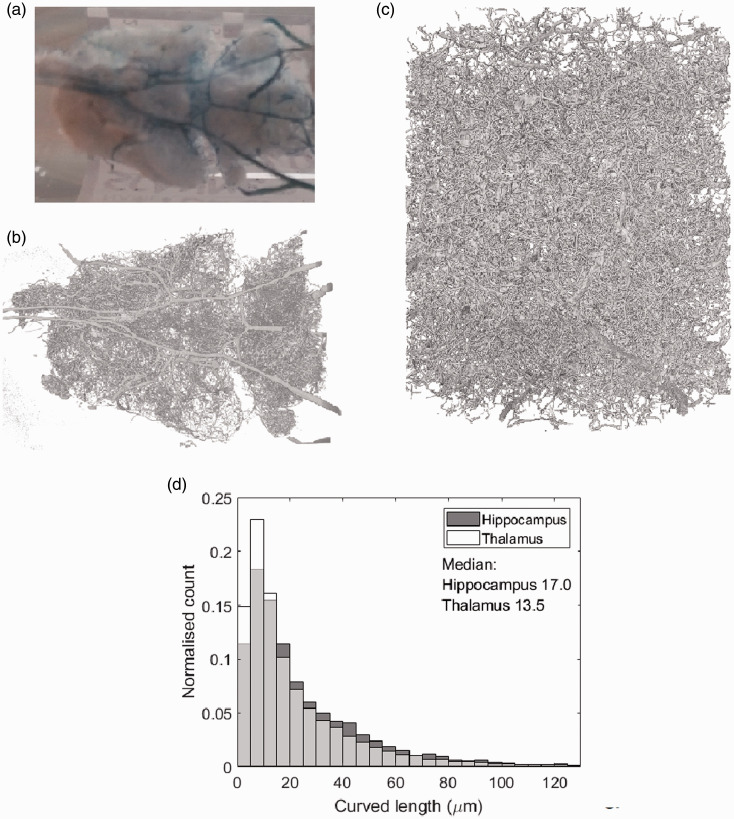
(a) A corrosion cast of a WKY rat – the circle of Willis is evident in blue. (b) and (c) show the volume render of reconstructed low resolution and high resolution µCT scans of the cast in (a) respectively. (d) shows the histogram of the capillary segment length l from the hippocampus and thalamus high resolution µCT scans. The median of each histogram is displayed in µm. The thalamus has a higher number of lower capillary segment lengths than the hippocampus- reflected in its lower median length of 13.5 µm compared to 17.0 µm.

To validate MR measures, corrosion casting was used to capture the vascular morphology of a single WKY rat aged 13 months. The animal was anaesthetised using isofluorane and Mercox (Ladd Research, Williston, VT) agent injected via transcardial perfusion. The left ventricle was held with a pair of haemostats and a 21 g catheter was inserted into the apex of the heart and clamped. The right atrium was severed and a pump started, running at 20 ml/min with 0.9% saline with heparin at 37 °C. 20 ml Mercox was prepared in a Leur lock syringe and slowly injected through the catheter into the heart. When perfusion was complete, the animal was stored overnight at 4 °C. The body was then removed, followed by a medial strip of the top of the skull– this was to maintain structural integrity of the cast during the dissolving of the tissue. Potassium hydroxide was used to dissolve tissue at 50 °C. The brain was rinsed numerous times with water and isopentane, then immersed in isopentane at −50°C to freeze. A photographic image of the corrosion cast is shown in Figure 3(a).

### Micro-CT of casts and analysis

To capture the fine resolution of microvessels, μ CT images were acquired of the corrosion cast. Large field of view (overview) scans were performed to facilitate positioning of higher spatial resolution ROI scans in the following brain regions: hippocampus, striatum, thalamus, and hypothalamus. Regions of interest were chosen by comparing the large vessel vascular architecture and structure of the reconstructed overview scan with the Paxinos structural rat atlas.^35^ Scans were acquired using a Zeiss Xradia Versa 520 3 D X-ray microscope (Zeiss, Oberkochen, Germany). Overview imaging, was performed using a magnification of x0.39, with a voltage of 70 kV and power of 6 W. Exposure time was set to 1 s, and 3201 projections were taken. The source-to-sample and sample-to-detector distances were 37.00 mm and 60.00 mm respectively. A binning of 2 was used, resulting in a cubic voxel of length 8.3 µm in the final reconstructed volume (Figure 3(b)). High resolution imaging used a magnification of x4, with a voltage of 80 kV and power of 7 W. Exposure time was set to 18 s, and 3201 projections were taken again. The source-to-sample and sample-to-detector distances were 25.01 mm and 87.65 mm respectively. A binning of 2 was used, resulting in a voxel size of 0.7499 µm^3^ in the reconstructed volume (Figure 3(c)). The scanned volume for the high resolution image was 1.499 mm^3^.

The original radiographs were reconstructed using filtered-back projection using the native Zeiss reconstructor software. A random subsample was selected from the high resolution dataset to allow practical data analysis. The subsample was chosen such that the largest vessels were avoided since these vessels were segmented less accurately than the smaller vessels. Approximately one quarter of the volume was chosen. The reconstructed 3 D volume was segmented and skeletonised using Avizo software (Thermo Fisher Scientific, Waltham, Massachusetts, USA). The semi-automated segmentation was done using a user controlled threshold limit above which voxels were binarised to one, and below which voxels were binarised to zero. The threshold limit was chosen based on the structures highlighted in a preview window – the ideal threshold would separate corrosion cast and background completely. However, noise meant this was not possible and a threshold was chosen with as few background voxels binarised to one as possible whilst still highlighting vessels. The segmentation was corrected by hand where the intravascular space was incorrectly binarised as background.

For each region, microvessel segment lengths were determined from the skeletonised data using the *‘auto skeleton’* function (example histogram in Figure 3(d)). The ‘auto skeleton’ function computes a skeleton and a spatial graph, which consists of nodes and segments, from which segment length is automatically estimated. A *‘spatial graph filter’* is applied which removes nodes which have more than four adjoining nodes since this was deemed unphysiological and was likely a result of segmentation error.

### Statistical analysis

Before estimating summary statistics of parameter estimates, outliers were removed by excluding those outside three median absolute deviations of the median. This method was chosen rather than the standard deviation since it is not affected by large outliers.^36^ Following outlier removal, the mean parameter estimate for each region for the two strains were calculated with standard error. The following analysis was performed to estimate the accuracy (bias) and repeatability (precision) in estimates from each model.

#### Comparison of MR with micro-CT estimates of capillary segment length

To assess accuracy and to validate the MR measurement of capillary segment length, the estimates were compared to those from the corrosion cast (ground truth) measure. The median vessel segment length of each region estimated from the μ CT data was correlated against the rat average MR estimate obtained using the VA, IVIM ballistic and VAB models. Linear regression using ‘*polyfit*’ in Matlab (R2018a) was used to test the association between the μ CT and MR estimates of l , returning the Pearson's correlation coefficient ρ , the associated p -value and the sum of squared errors (SSE).

#### Assessment of scan-rescan repeatability

A subset of five F344 rats underwent repeat measurements within two weeks of first measurement. The coefficient of variation (CoV) was calculated for each region to investigate repeatability of the measures and analysis methods:

(11) $$\begin{array}{c} \text{C}\text{o}\text{V}\text{(}\text{\%}\text{)}=100\frac{\sigma }{\mu } \end{array}$$

where σ is the within subject standard deviation and μ the mean. σ is calculated using:^37^

(12) $$\begin{array}{c} \sigma =\sqrt{\frac{{{\sum (x}_{1}-x_{2})}^{2}}{2n}} \end{array}$$

where n is the number of repeated measures, $x_{i}$ is the parameter estimate for each subject at scan i . μ is found using:

(13) $$\begin{array}{c} \mu =\frac{\sum \left(x_{1}+x_{2}\right)}{2n} \end{array}$$

#### Comparison of MR estimates of velocity, v , and capillary segment length,  l between rat strains

To assess agreement of measurements between two rat strains, expected to have similar regional variation, the regional averages of v and l were compared between F344 and WKY rats for the VA and VAB models. Pearson’s correlation coefficient, ρ , and the associated p -value were estimated, as well as the regression equation.

The Anderson-Darling test was used to assess normality – the *‘adtest’* function was used in Matlab (R2018a). Approximately 80% of data groups had a normal distribution, hence a repeated measures two-way ANOVA was performed for $D^{*}$ , v and l to assess inter-strain and inter-regional differences. The test was performed for the VA and VAB models only using the *‘ranova’* function in Matlab (R2018a).

#### Comparison of voxel-wise SNR between diffusion-times

A repeated measures two-way ANOVA was also performed for voxel-wise SNR to assess inter-regional and inter-diffusion-time differences.

## Results

### Comparison of MR and CT estimates of capillary segment length

**Figure 4.**
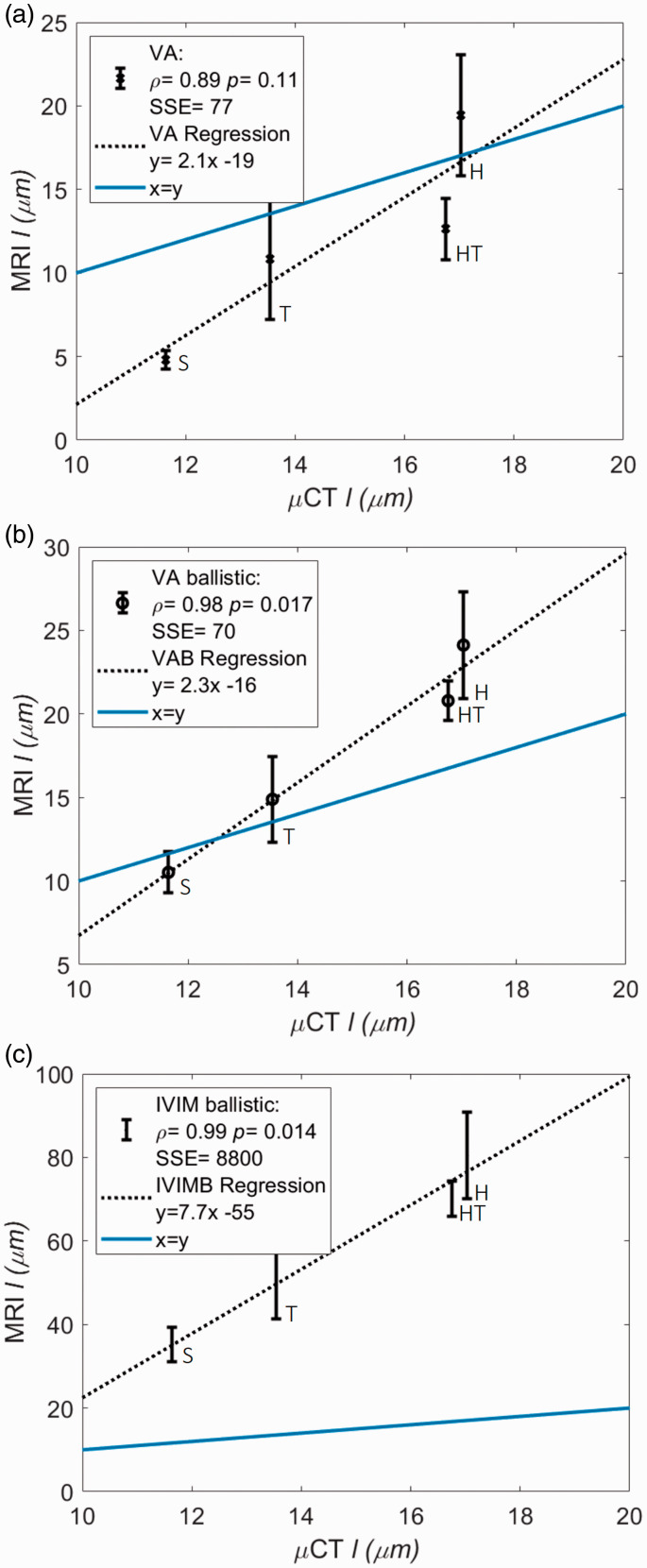
Regional average WKY MRI estimates of l (± standard error of the mean) versus the median of μ CT estimates. Estimates are shown from three methods of velocity ( v ) estimation which then uses $D^{*}=vl/6$ to estimate l : the VA model (a), the VA ballistic (VAB) model (b) and the IVIM ballistic (IVIMB) model (c). The line of identity is displayed (blue), as is the regression line for each method (dashed black). Estimates for the striatum (S), thalamus (T), hypothalamus (HT) and hippocampus (H) are shown.

Figure 4 shows the μ CT versus MR estimates for the VA, VAB and IVIM ballistic models in parts a, b and c respectively. The VAB model showed the greatest level of agreement with μ CT estimates of capillary segment length (i.e. lowest bias indicated by the lowest sum of squared errors SSE = 70 and least negative y-intercept in the regression equation (y(x = 0) = −16)), although the VA model had similar levels of agreement (SSE = 77, y(x = 0) = −19). The IVIM ballistic model had substantially poorer agreement (SSE = 8800 and y(x = 0) = −55), with approximately 3.5-fold higher estimates of l than the VAB model. All models produced estimates of l that were highly correlated with μ CT estimates ( ρ>  0.89 for all models). The linear regression between μ CT and MR estimates was significant only for the VAB and IVIM ballistic models, with p = 0.017 and p = 0.014 respectively. Evidence for a linear relationship between VA model estimates of l and μ CT was low ( p = 0.11). Since estimates using the IVIM ballistic model are inaccurate only the two VA models will be compared henceforth.

### Assessment of scan-rescan repeatability

The CoV of parameters are shown in Table 1. The CoV of vascular parameters ( f , $D^{*},\;v$ , l ) was highest in the frontal cortex, and lowest in the striatum. Averaged across regions, scan re-scan CoVs were 32.0% for f and 58.0% for $D^{*}$ . Extravascular D had a region-averaged CoV of 3.70%, much lower than that of vascular parameters, likely due to the much higher signal fraction of extravascular water. The CoV for the VAB model was the lowest of the two methods, with average values of 22.4% for v  and 73.1% for l . The CoV of the VA model had average values of 45.8% for v and 109% for l .

**Table 1.** Coefficient of variation (%) of parameter estimates calculated from repeat measures. Results from five of the F344 strain.

	IVIM diffusive			VA		VA ballistic	
Region	f	D	$D^{*}$	v	l	v	l
Striatum	10.2	1.84	13.0	39.3	79.1	16.8	32.2
Temporal cortex	41.1	5.18	118	59.4	137	19.4	126
Cingulate cortex	19.2	1.55	18.4	20.9	25.0	26.7	54.2
Entorhinal cortex	33.2	3.40	70.2	36.2	147	22.4	85.6
Frontal cortex	72.9	12.8	95.9	81.8	245	25.7	130
Motor cortex	46.3	2.37	66.3	43.8	121	44.5	76.6
Parietal cortex	11.6	0.508	27.4	50.8	70.0	27.3	28.2
Hippocampus	37.1	3.86	80.8	16.5	97.0	13.4	82.3
Hypothalamus	33.1	4.67	21.2	46.0	64.6	8.77	26.3
Thalamus	19.4	2.37	69.0	63.3	110	19.5	90.2
**Mean**	**32.0**	**3.70**	**58.0**	**45.8**	**109**	**22.4**	**73.1**

f : perfusion fraction; D : diffusion coefficient; $D^{*}$ : pseudo-diffusion coefficient (all from the IVIM diffusive model fit); v : average blood velocity (from the VA and VA ballistic models); l : capillary segment length (from $D^{*}=vl/6$ ).

### Comparison of VA and VA ballistic MR estimates of velocity, v , and capillary segment length,  l between rat strains

**Figure 5.**
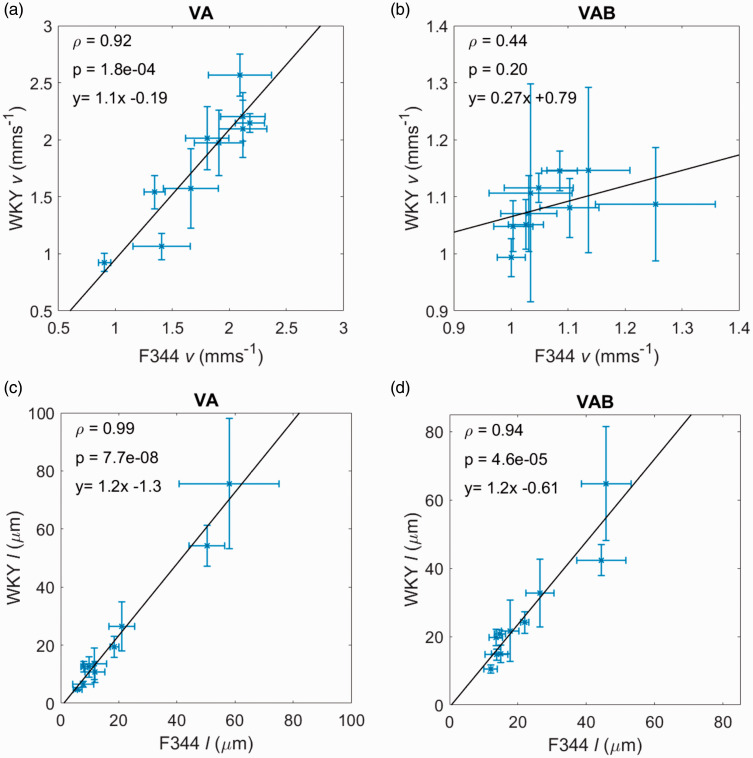
Regional averages (± standard error of the mean) of velocity ( v ) and capillary segment length ( l ) for the two rat strains (F344 and WKY) for two different methods of parameter estimation: (a) and (c) for the VA model; and (b) and (d) for the VA ballistic model. Pearson’s correlation coefficient, ρ is shown as well as the p -value which tests the hypothesis that there is no relationship between the observed parameters. Each point represents a different region.

Regional average values for v and l for the F344 and WKY rats are shown in Figure 5 for the VA and VAB models. Each point represents a different region of the brain (these values are also displayed in Tables S1 and S2 in the supplementary material, alongside estimates of the perfusion fraction, f , diffusion coefficient, D , and pseudo-diffusion coefficient, $D^{*}$ ). f and D  are in good agreement with literature values.^31^,^32^

Generally, across both strains, $v_{\text{VA}}\;$ was greater than $v_{\text{VAB}}$ resulting in values for l that were slightly lower for the VA model, with exception of the cingulate cortex for both the F344 and WKY strains, and the frontal cortex for the WKY strain. l was lowest for the striatum across both methods of estimation and both rat strains. l was highest for the frontal cortex. Variability of estimates between rats was also much larger in the frontal cortex than in other regions, as indicated by the generally larger standard error. This variability is likely due to poor measurement precision, as indicated by the poor scan-rescan repeatability in this region. Values for $D^{*}$ were higher than other regional values for the frontal cortex which could explain large values for l . The mean values for v and l can be found in the supplementary material for each region, model and strain (Tables S1 and S2). Outlier removal for v and $D^{*}$ are shown in Table S3.

Estimates of the blood velocity, v , were in good agreement between the strains – region average $v_{\text{VA}}$ was equal to 1.77 mms^−1^ and 1.86 mms^−1^, and $v_{\text{VAB}}$ equal to 1.07 mms^−1^ and 1.08 mms^−1^, for the F344 and WKY strains respectively. l varied between strains for the two models due to differing $D^{*}$ . The correlation of parameter estimates between rat strains was highest for the VA model for both parameters ( $\rho_{v}$ = 0.92 and $\rho_{l}$ = 0.99). The p -values of the linear regressions were also lowest for this model $(p_{v}$ = 1.8 × 10^−4^ and $p_{l}$ = 7.7 × 10^−8^), reflecting the ability of the VA model to estimate a wider dynamic range of parameters, particularly for  v . The regression equations for $v_{VA}$ and $l_{VA}$ show low bias and excellent agreement between strains, as does the equation for $l_{\mathrm{VAB}}$ . The equation for $v_{\mathrm{VAB}}$ indicates less agreement between strains, although averages agree well, with a low gradient due to $v_{\mathrm{WKY}}>v_{F344}$ .

Two-way repeated measures ANOVA tests (Table S4) showed no significant effects of genotype on $D^{*}$ , v or l for the VA and VAB models, reflecting the similar parameter values between rat strains. The effects of region were significant for the VA model only ( $p_{v}$ = 0.029 and $\;p_{l}$ = 1.8 × 10^−5^), again reflecting the wider dynamic range of parameter estimates than those of the VAB model. The region and genotype interaction had no significant effects on any parameters.

### Assessment of model validity

**Figure 6.**
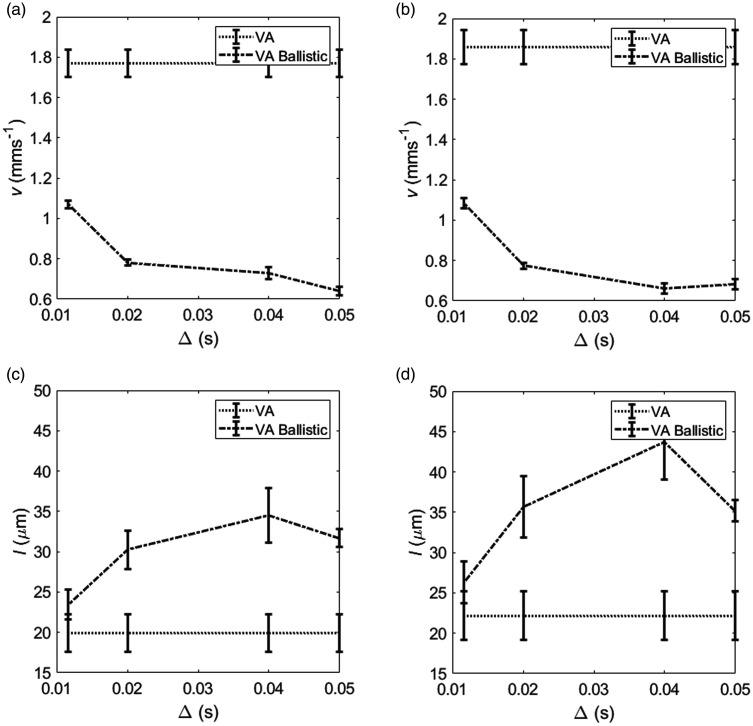
(a) and (c) show F344 rat and region average (± standard error of the mean) for estimates of the velocity, v , and capillary segment length,  l , for the velocity autocorrelation model (VA) and the VA ballistic model. l is calculated using the pseudo-diffusion coefficient ( $D^{*}$ ) estimated using Δ =50 ms. (b) and (d) show the same for the WKY rat strain. The points are joined to show the relative values for each model more clearly. The values for the VA model are flat since all diffusion-times are used in parameter estimation.

Since the VAB model is a simplification of the VA model, valid where $T_{0}>\Delta$ , estimates of v should converge to the VA estimate at lower diffusion-times. Figure 6(a) and (b) showed the beginning of this convergence, however, the estimates of v did not meet. This suggests the diffusion-time of 11.6 ms used in this study is not low enough for ballistic model validity (i.e. $\;T_{0}>\Delta$ is not met) and v may be underestimated. The same trend occurred with l , although the estimates at 11.6 ms were much closer with only a small difference in l (Figure 6(c) and (d)).

### Signal to noise ratio

The voxel-wise SNR for each region and diffusion-time at b = 10 smm^−2^ are shown in Supplementary Table S5. The voxel-wise SNR does not exhibit large variation across diffusion-times, however, the frontal cortex and motor cortex generally have lower SNR than other regions. This does not appear to have affected parameter estimates for the motor cortex, since the CoV and parameter estimates are comparable to other regions. However, parameter estimates and CoV in the frontal cortex are larger than those of other regions.

A two-way repeated measures ANOVA test showed a significant effect of region on SNR ( p = 9.48 × 10^−5^), but no significant effect of diffusion-time ( p = 0.203).

## Discussion

In this study, we use multi-diffusion-time DW-MRI to compare models of vascular flow across different blood flow regimes (Figure 1) for estimating v and l . Through validating MR estimates against μ CT measurements (Figure 3) and assessing scan-rescan repeatability and inter-strain comparisons, we show the VAB model has the highest accuracy (Figure 4) and precision (Table 1) in estimating l . While we are unable to ascertain the accuracy of v , we observe that the VAB model also provides high repeatability for this parameter. Results also highlight the potential of the VA model, with reasonable repeatability for v , high correlation between rat strains for both parameters and comparable accuracy to the VAB model.

Comparing MR with μ CT estimates (Figure 4) suggests that the VAB model produces the most accurate estimates of l . Although, as indicated by the low SSE, estimates made by the VA model also show low levels of bias. VAB estimates are generally higher than μ CT estimates, whereas VA estimates are generally lower – the lowest being on the order of literature values for vessel diameter.^34^,^38^,^39^ The IVIM ballistic model produced estimates approximately 3.5 times larger than μ CT estimates.

Scan-rescan repeatability data (Table 1) suggests that the VAB model gives more precise estimates of v and l . These high levels of precision could be due to the inability of the VAB model to capture variability, as indicated by the low regional range in $v_{\text{VAB}}$ (Figure 5). Although this is difficult to determine, since values for v in literature vary substantially and information regarding regional variation is scarce. We speculate that high values for CoV for the frontal cortex are due to inclusion of some non-brain voxels due to segmentation errors, but may also be due to susceptibility artefacts or the lower voxel-wise SNR in this region (Table S5 in supplementary material).

Values of $v_{\text{VA}}$ and $l_{\text{VA}}$ (Figure 5) had highest correlation between the two rat strains, indicating the VA model may be more sensitive to regional variability – the VAB model generally produces estimates within a smaller range. ANOVA results confirm this, with region having a significant effect on $v_{VA}$ but not $v_{\mathrm{VAB}}$ . The strong correlation in $l_{\text{VAB}}$ between the two strains could be driven by $D^{*}$ - this would also explain why the pattern of results is similar between the VA and VAB models (Figure 5(c) and (d)). Both models produce a comparable number of spurious values (i.e. outliers, see Table S4).

Estimates of v in the literature vary substantially. Unekawa et al.^34^,^40^ used laser-scanning confocal microscopy with a high speed camera to measure a mean red blood cell (RBC) velocity in cortical capillaries of 2.05 ± 1.6 mms^−1^. Masamoto et al.^41^ measured mean RBC velocity in cortical microcirculation to be 1.5 ± 0.4 mms^−1^. These values are in agreement with some of our regional values of $v_{\text{VA}}$ . Unekawa et al.^40^ also present results from previous studies, with a variety of methods, where the mean across studies is 0.86 ± 0.47 mms^−1^, which is closer to estimates from the VAB model. The variation between studies possibly comes from differing definitions of capillaries, through which v is likely to vary – for example, depending on the study, capillaries have been defined previously as vessels with diameter <5 µm,^42^ <6 µm,^43^ <8 µm^41^ and <10 µm^40^. Differing anaesthesia will also affect RBC velocity estimates – Masamoto et al.^41^ show a mean velocity of 0.4 ± 0.4 mms^−1^ and 1.5 ± 0.4 mms^−1^ for α-chloralose and isoflurane respectively.

Although estimates of l herein agree with the range of results presented in literature,^44–46^ the estimates are generally lower than average values presented in the same papers. Bosomtwi et al.^33^ present a method of measuring l *in-vivo*^47^ in rats with embolic stroke. The paper estimates l = 26.52 ± 3.20 µm in the contralateral region. An immunohistochemical approach presented by Morris et al.^48^ estimate l in Wistar rats to be around 27 µm. These estimates are comparable to our region average $l_{\text{VAB}}$ , however a few regions, such as the striatum and motor cortex, have values of around half these estimates. Large estimates for length scale are presented by Mironov et al.^44^ of 68.9 ± 4.5 µm in the superficial cortex of rats, which would agree with only the largest estimates of $l_{VA}$ presented. Mirinov’s result is the branch length obtained from corrosion casts, which differs greatly from both our MR and μ CT estimates.

The VAB model is a simplification of the VA model and the two should converge where the ballistic regime ( $T_{0}>\Delta )$ is valid. Figure 6 shows that this assumption is not necessarily met at the shortest diffusion-time of 11.6 ms with the VAB model underestimating v and overestimating l compared to the VA model. This could be due to blood flow changing direction when the model assumes no changes. Protons are distributed evenly across the length of the vessel segment at the onset of diffusion-sensitising gradients, so whilst all protons may travel the same distance, some will change segment and so direction during the pulse application time. This will result in a shorter apparent distance travelled and so reduced estimated velocity. If diffusion-time could be lowered by using stronger diffusion gradients, the VAB model and VA model should in theory produce similar values for v . This would be difficult to achieve on a clinical scanner, unless oscillating gradients were used.^15^

Studies using flow-compensated DWI have shown that the diffusive regime ( $T_{0}<\Delta )$ is not met at diffusion-times similar to those used in this study (50 ms).^21^,^23^ This may suggest that estimates of $D^{*}$ are biased. However, since values for l  generally compare well with literature values and corrosion cast estimates, it appears that the effect of these biases are minor, or may cancel. Studies using flow-compensated DWI allow for extraction of v and, with the method proposed by Wetscherek et al.,^23^ ${\;T}_{0}$ , therefore giving l . For our data set, $T_{0}$ was highly variable, had low repeatability and a large number of outliers, so was not used to estimate l (results not shown). This was likely due to the restricted number of diffusion-times. The flow-compensated method increases sensitivity to flow in capillaries by reducing signal contribution from larger vessels where blood does not change direction. However, estimates by Wetscherek et al.^23^ in the liver and pancreas suggest v is higher than expected in capillaries, which would lead to overestimation of  l . Ahlgren at al.^21^ produce estimates of v closer to those expected in brain capillaries when using different assumptions about the phase distribution to Wetscherek. Increased imaging times and sensitivity to eddy currents mean that our proposed method may be more appropriate to clinically implement - although Gurney-Champion et al.^22^ do show that a clinically feasible protocol is possible with flow-compensated gradients, but the protocol performs better in highly perfused tissues.

One general limitation of our method is the need to collect data at multiple *b*-values and diffusion-times leading to long acquisition times or relatively sparse sampling of the parameter space. In this study, this resulted in a small number of b -values in the intravascular range, meaning it was not possible to investigate the two-compartment intravascular models which have recently been proposed in IVIM studies. However, the monoexponential intravascular model fits well and the estimated length scales correlated well with ground truth measures from μ CT of a corrosion cast, supporting the model. In addition, while biexponential modelling of the vascular signal may improve the accuracy of vessel segment length estimation, the precision of estimates may worsen. Regardless of the IVIM model used, assumptions made when developing biophysical models generally mean underlying biological processes are not fully characterised, which may result in inaccurate parameter estimates. For example, in the diffusive regime, $D^{*}$ models blood flow in the microvasculature as Brownian motion. Additionally, all models of IVIM assume zero water exchange between compartments. In reality, both of these assumptions may break down.

In addition, the limited range of diffusion-times used could affect accuracy of parameter estimates – although, estimates of $D^{*}$ and v were very reasonable when compared with the literature. This, along with the μ CT data, suggests estimates of l are also reasonable. While μ CT imaging of corrosion casts provided a means to measure capillary segment length directly, it is important to note that a number of factors can lead to inaccuracies including: incomplete perfusion of the resin, potential change in shape of the cast during transit (although this was mitigated by the skull remaining intact when dissolving tissue and drying the cast) and possible cast damage when tissue is dissolved. Approximations made when reconstructing μ CT data computationally may also introduce inaccuracies. Finally, we only had access to one corrosion cast from one rat strain (WKY) due to difficulties with the casting process. This clearly limits our ability to comprehensively validate the MRI measurements.

## Conclusion

In this study, we determined that the ballistic approximation of the velocity autocorrelation (VAB) model applied in combination with the diffusive IVIM model to low and high diffusion-time data provides the most accurate and precise estimates of capillary segment length. The length scales estimated using these models correlated well with μ CT measures of a vascular corrosion cast. Results from the VA model were encouraging, with good agreement with μ CT estimates of l , however estimates were less repeatable according to CoV calculations. Inter-strain comparisons of v and l  suggest that the VA model accurately captures regional variability in v across rat strains. The ballistic IVIM model produced substantially biased estimates of l . We have shown that multi-diffusion-time DW-MRI is able to estimate capillary blood velocity and segment length with reasonable accuracy and precision.

## Supplemental Material

sj-pdf-1-jcb-10.1177_0271678X20978523 - Supplemental material for Characterisation of microvessel blood velocity and segment length in the brain using multi-diffusion-time diffusion-weighted MRI Click here for additional data file. Supplemental material, sj-pdf-1-jcb-10.1177_0271678X20978523 for Characterisation of microvessel blood velocity and segment length in the brain using multi-diffusion-time diffusion-weighted MRI by Lauren A Scott, Ben R Dickie, Shelley D Rawson, Graham Coutts, Timothy L Burnett, Stuart M Allan, Geoff JM Parker and Laura M Parkes in Journal of Cerebral Blood Flow & Metabolism
