# Supplementary material for: Characterisation of microvessel blood velocity and segment length in the brain using multi-diffusion-time diffusion-weighted MRI
Source: J Cereb Blood Flow Metab. 2020 Dec 16;41(8):1939–53. doi: 10.1177/0271678X20978523 (PMC8323340; doi:10.1177/0271678X20978523)
Supplement: sj-pdf-1-jcb-10.1177_0271678X20978523 - Supplemental material for Characterisation of microvessel blood velocity and segment length in the brain using multi-diffusion-time diffusion-weighted MRI [file sj-pdf-1-jcb-10.1177_0271678X20978523.pdf]

Table S1: F344 rat averages of the perfusion fraction (f), diffusion coefficient (D), pseudo-diffusion coefficient (D^*), average velocity (v) and average capillary segment length (l) for two models (VA – velocity autocorrelation model; and VA ballistic) with standard error of the mean. l is calculated using the relationship $D^* = vl/6$.							
	IVIM diffusive			VA		VA Ballistic	
Region	f	D x 10^{-4} (mm^2s^{-1})	$D*$ x 10^{-3} (mm^2s^{-1})	v (mms^{-1})	l (μm)	v (mms^{-1})	l (μm)
Striatum	0.0501 ± 0.0039	6.47 ± 0.046	1.90 ± 0.23	2.18 ± 0.13	5.83 ± 1.4	1.00 ± 0.025	12.0 ± 2.0
Temporal Cortex	0.0340 ± 0.012	6.73 ± 0.18	4.45 ± 0.73	1.66 ± 0.24	21.0 ± 4.4	1.03 ± 0.073	26.5 ± 4.1
Cingulate Cortex	0.0877 ± 0.0070	6.45 ± 0.064	6.65 ± 0.64	0.906 ± 0.054	50.3 ± 6.2	1.03 ± 0.049	44.4 ± 7.2
Entorhinal Cortex	0.0448 ± 0.0096	7.03 ± 0.14	2.82 ± 0.37	2.12 ± 0.21	11.7 ± 4.1	1.14 ± 0.073	17.6 ± 2.6
Frontal Cortex	0.0614 ± 0.018	7.05 ± 0.22	7.36 ± 1.1	1.41 ± 0.25	58.0 ± 17	1.25 ± 0.10	45.8 ± 7.2
Motor Cortex	0.0396 ± 0.0089	6.62 ± 0.045	1.93 ± 0.59	2.09 ± 0.28	7.76 ± 3.6	1.05 ± 0.060	13.7 ± 3.3
Parietal Cortex	0.0380 ± 0.0056	6.647± 0.065	2.38 ± 0.28	1.91 ± 0.21	9.74 ± 1.9	1.03 ± 0.031	13.5 ± 2.0
Hippocampus	0.0612 ± 0.010	7.13 ± 0.16	3.64 ± 0.23	1.34 ± 0.094	18.5 ± 1.5	1.10 ± 0.052	21.9 ± 1.2
Hypothalamus	0.0793 ± 0.011	6.73 ± 0.097	2.51 ± 0.27	2.11 ± 0.20	8.02 ± 1.1	1.09 ± 0.031	14.7 ± 1.7
Thalamus	0.0587 ± 0.010	6.44 ± 0.13	2.46 ± 0.46	1.81 ± 0.19	11.8 ± 3.4	1.00 ± 0.034	15.0 ± 2.8
**Mean ± std error**	**0.0522 ± 0.0020**	**6.75 ± 0.032**	**3.64 ± 0.24**	**1.77 ± 0.068**	**19.9 ± 2.3**	**1.07 ± 0.018**	**23.5 ± 1.9**

Table S2: WKY rat averages of the perfusion fraction (f), diffusion coefficient (D), pseudo-diffusion coefficient (D^*), velocity (v) and capillary segment length (l) for two models (VA – velocity autocorrelation model; and VA ballistic) with standard error of the mean. l is calculated using the relationship $D^* = vl/6$.							
	IVIM diffusive			VA		VA ballistic	
Region	f	D x 10^{-4} (mm^2s^{-1})	$D*$ x 10^{-3} (mm^2s^{-1})	v (mms^{-1})	l (µm)	v (mms^{-1})	l (µm)
Striatum	0.0559 ± 0.0041	6.40 ± 0.055	1.82 ± 0.19	2.15 ± 0.082	4.80 ± 0.55	0.994 ± 0.033	10.5 ± 1.2
Temporal Cortex	0.0568 ± 0.0070	6.68 ± 0.073	4.80 ± 1.2	1.57 ± 0.35	26.5 ± 8.5	1.11 ± 0.19	32.7 ± 9.9
Cingulate Cortex	0.108 ± 0.0051	6.38 ± 0.081	7.52 ± 0.68	0.924 ± 0.079	54.2 ± 7.0	1.07 ± 0.067	42.4 ± 4.5
Entorhinal Cortex	0.0524 ± 0.0066	6.86 ± 0.11	2.99 ± 0.74	2.09 ± 0.25	13.6 ± 5.4	1.15 ± 0.14	21.7 ± 9.0
Frontal Cortex	0.0727 ± 0.020	6.94 ± 0.34	10.1 ± 2.5	1.06 ± 0.12	75.6 ± 22	1.09 ± 0.099	64.8 ± 17
Motor Cortex	0.0539 ± 0.0045	6.70 ± 0.084	2.66 ± 0.33	2.57 ± 0.18	6.52 ± 0.94	1.12 ± 0.026	14.8 ± 1.6
Parietal Cortex	0.0483 ± 0.0032	6.60 ± 0.059	3.54 ± 0.21	1.97 ± 0.29	12.5 ± 3.5	1.05 ± 0.044	19.8 ± 2.4
Hippocampus	0.0824 ± 0.0057	6.95 ± 0.055	4.31 ± 0.43	1.54 ± 0.15	19.4 ± 3.6	1.08 ± 0.051	24.1 ± 3.2
Hypothalamus	0.112 ± 0.0073	6.95 ± 0.10	3.97 ± 0.26	2.20 ± 0.21	12.6 ± 1.8	1.15 ± 0.035	20.8 ± 1.2
Thalamus	0.0645 ± 0.0047	6.40 ± 0.10	2.62 ± 0.35	2.01 ± 0.28	10.8 ± 3.6	1.05 ± 0.045	14.9 ± 2.6
**Mean ± std error**	**0.0648 ± 0.00038**	**6.73 ± 0.029**	**4.48 ± 0.39**	**1.86 ± 0.068**	**22.2 ± 3.0**	**1.08 ± 0.027**	**26.3 ± 2.6**

Table S3: Percentage outliers removed over all regions for each parameter and strain. D^* is the pseudo diffusion coefficient and v is average blood velocity, from the velocity autocorrelation (VA) and VA ballistic models.						
	F344			WKY		
	D^*	v		D*	v	
Region	IVIM diffusive	VA	VA Ballistic	IVIM diffusive	VA	VA Ballistic
Striatum	13	16	5.9	20	10	0
Temporal Cortex	7.1	5.9	24	10	20	0
Cingulate Cortex	19	11	0	0	10	0
Entorhinal Cortex	23	0	0	0	0	0
Frontal Cortex	8.3	13	0	0	25	22
Motor Cortex	20	0	12	0	0	22
Parietal Cortex	12	0	12	30	0	11
Hippocampus	29	12	0	0	10	0
Hypothalamus	25	0	12	10	0	0
Thalamus	25	0	0	10	0	0
**Mean**	**18**	**5.8**	**6.6**	**8**	**7.5**	**5.5**

Table S4: Repeated-measures two-way analysis of variance (ANOVA) p-values for the pseudo-diffusion coefficient (D^*), average blood velocity (v) and the average capillary segment length (l) for the velocity autocorrelation (VA) and VA ballistic models.					
	D^*	v		l	
Factors	IVIM diffusive	VA	VA ballistic	VA	VA ballistic
Genotype	0.073	0.51	0.98	0.21	0.055
Region	0.054	0.029	0.14	1.8×10^{-5}	0.11
Region*Genotype	0.65	0.48	0.38	0.056	0.96

Table S5: Voxel-wise signal to noise ratio (SNR) for four diffusion-times at b=10 smm^{-2}, averaged across F344 rats using the mean (± standard error of the mean).				
	Diffusion-time (ms)			
Region	50	40	20	11.6
Striatum	13.8 ± 0.18	14.4 ± 0.11	14.6 ± 0.15	14.4 ± 0.24
Temporal cortex	12.3 ± 1.0	11.0 ± 1.3	12.3 ± 1.3	13.2 ± 1.4
Cingulate cortex	14.2 ± 0.31	17.7 ± 0.50	17.4 ± 0.30	17.3 ± 0.68
Entorhinal cortex	11.3 ± 0.73	11.0 ± 0.24	12.9 ± 0.45	11.7 ± 0.75
Frontal cortex	6.17 ± 0.92	7.46 ± 0.64	7.38 ± 0.92	7.73 ± 0.77
Motor cortex	2.80 ± 0.32	8.58 ± 0.29	8.08 ± 0.88	7.79 ± 0.93
Parietal cortex	9.50 ± 0.75	13.3 ± 0.98	11.6 ± 0.56	11.8 ± 0.97
Hippocampus	14.9 ± 0.34	14.2 ± 0.29	15.1 ± 0.32	15.7 ± 0.43
Hypothalamus	13.1 ± 0.28	13.3 ± 0.69	13.4 ± 0.73	14.4 ± 0.77
Thalamus	14.2 ± 0.34	14.0 ± 0.31	13.8 ± 0.19	14.3 ± 0.46

Animal breeding, housing, and husbandry

Fischer 344 rats were purchased from Terrence Town labs at the University of Southern California and then aged in-house. Wistar-Kyoto rats were purchased from Janvier Labs, France, and aged in-house. Throughout their lifetime, animals were housed in a conventional rodent facility in individually ventilated cages (between 3-4 animals per cage). The facility had a 12/12 hour dawn/dusk cycle, beginning at 7.30 am. Bedding was aspen woodchip and paper sizzle nest. Animals were given the following environmental enrichment: sizzle nest, play tunnels, aspen bricks (chew sticks) and aspen balls. Housing temperature and humidity were maintained at 19-23°C and 45-65% respectively, conforming to the Home Office code of practice for housing rodents. All animals were fed ad libitum on the BK001(E) diet (standard diet) and given free access to water. Animal health was checked daily and respiratory problems assessed prior to anaesthesia. After MRI, animals were recovered until they were able to take food and water, then returned to housing.
